# Lipid Droplets and Their Participation in Zika Virus Infection

**DOI:** 10.3390/ijms232012584

**Published:** 2022-10-20

**Authors:** Zhao-Ling Qin, Qiu-Feng Yao, Hao Ren, Ping Zhao, Zhong-Tian Qi

**Affiliations:** Department of Microbiology, Naval Medical University, Shanghai 200433, China

**Keywords:** lipid droplets, Zika virus, lipid metabolism, lipophagy, replication, capsid protein

## Abstract

Lipid droplets (LDs) are highly conserved and dynamic intracellular organelles. Their functions are not limited to serving as neutral lipid reservoirs; they also participate in non-energy storage functions, such as cell lipid metabolism, protection from cell stresses, maintaining protein homeostasis, and regulating nuclear function. During a Zika virus (ZIKV) infection, the viruses hijack the LDs to provide energy and lipid sources for viral replication. The co-localization of ZIKV capsid (C) protein with LDs supports its role as a virus replication platform and a key compartment for promoting the generation of progeny virus particles. However, in view of the multiple functions of LDs, their role in ZIKV infection needs further elucidation. Here, we review the basic mechanism of LD biogenesis and biological functions and discuss how ZIKV infection utilizes these effects of LDs to facilitate virus replication, along with the future application strategy of developing new antiviral drugs based on the interaction of ZIKV with LDs.

## 1. Introduction

Zika virus (ZIKV) is a mosquito (*Aedes*)-borne human pathogen that spreads via human bodily fluids. The viral infection causes fever, rash, headache, joint and muscle pain, conjunctivitis, severe eye lesions, newborn microcephaly, and Guillain–Barré syndrome [1,2,3,4,5,6]. The virus has been transmitted and has spread rapidly in the Americas, especially in Brazil [7]; the World Health Organization (WHO) declared it a global public health concern for public health on 1 February 2016. The viral infection is also likely to spread to many uninfected countries and regions due to the widespread distribution of the *Aedes* that transmits it, the frequent international trade activities, and rising tourism [8]. In addition, no vaccines or specific antiviral therapy have been approved to prevent or treat its infection. Nevertheless, researchers have been actively developing effective prevention and control strategies in response to the severe threat of ZIKV infection, which is a vital research goal.

**Figure 1:**
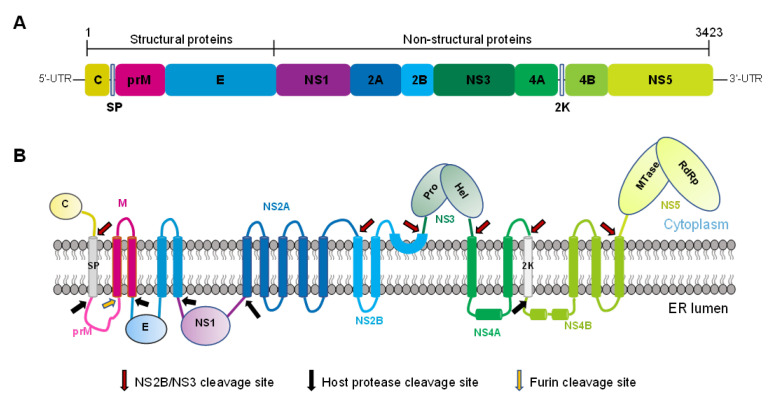
Schematic diagram of ZIKV polyprotein organization and processing. (**A**) Linear organization of the ZIKV polyprotein precursor, including the three structural and seven nonstructural proteins. (**B**) Topology of the polyprotein in the ER membrane and its sites of splicing by the host or viral proteases.

ZIKV belongs to the genus *Flavivirus* and the family *Flaviviridae* [9], which also includes viruses such as the dengue virus (DENV) and West Nile virus (WNV). Its genome is a single-stranded positive RNA with a length of about 11 kb, which encodes a single open reading frame and translates into a multi-protein precursor. This polyprotein is usually processed by the virus and host protease into three structural proteins, including the capsid ©, the pre-membrane (prM), and the envelope (E). The polyprotein is also processed into seven non-structural (NS) proteins, namely, NS1, NS2A, NS2B, NS3, NS4A, NS4B, and NS5, which participates in viral replication, pathogenesis, and host antiviral reactions (Figure 1) [6,10]. Lipidomics and transcriptomics analysis on the different ZIKV-infected mosquito cells, small glial cells, primary retinal pigmented epithelial (RPE) cells, or serum from patients indicate that the viral infection significantly regulates lipid metabolism, causing changes in a large number of lipids in cells. The changes in lipids include those to glycerophospholipids, such as phosphatidylcholine (PC) and phosphatidylserine, sphingolipids, such as ceramide and sphingomyelin, plasmalogens, and lysophospholipids, such as lysophosphatidylcholine [11,12,13,14]. These lipids may participate in the critical processes of ZIKV infection, such as genome replication and virion biogenesis. The envelope of ZIKV has been reported from the membranes of the host cells, while the association of the virus with the remodeling of lipid metabolism demonstrates that its successful infection depends on the host lipid metabolism.

Most of ZIKV’s structural and NS proteins possess transmembrane domains, allowing them to have specific locations on cellular organelles [9,10,15,16]. Other ZIKV proteins without transmembrane sequences, such as NS1, are also associated with the cell membrane [17], indicating that viral proliferation during the infection is closely related to the membranes of plasma and other cellular organelles to which the viral proteins bind. For example, the endoplasmic reticulum (ER) plays an essential role in ZIKV infection by undergoing substantial rearrangement after viral infection in cells such as C6/36 in mosquitoes, Vero, human hepatic cells (Huh7), and human neural progenitor cells (hNPCs) in mammals. The ER remodeling is related to virus infection because viral replication sites, assembly, and budding occur in the vicinity of the ER region [18,19,20]. The NS3 protease also needs the activation of the co-factor NS2B that anchors onto the ER membrane [21]. The NS2A protein is located in the peroxisome; its transient expression changes the organelle’s morphology and distribution, verifying the role of peroxisomes in ZIKV infection [16]. The analysis of the serum from patients infected with ZIKV showed that the levels of several types of phosphatidylethanolamine (PE) increased, and the synthesis of plasmalogens depended on functional peroxisome, which further supports the role of the lipids during ZIKV infection [13]. In addition, lipids are stored in cells as lipid droplets (LDs), in which the majority of the ZIKV C protein is found. This association is eliminated by substituting specific amino acids in the C-protein [22]. Although the consequences of this association have not yet been deciphered, it may be important for virion biogenesis, as described for hepatitis C virus (HCV) and DENV infections [23,24].

## 2. Basics of Lipid Droplets

Cellular LDs are highly conservative and dynamic intracellular organelles that are common in animals, plants, fungi, and bacteria [25,26] and fulfill a variety of functions, including storing neutral lipids in cells.

### 2.1. LDs Composition

**Figure 2:**
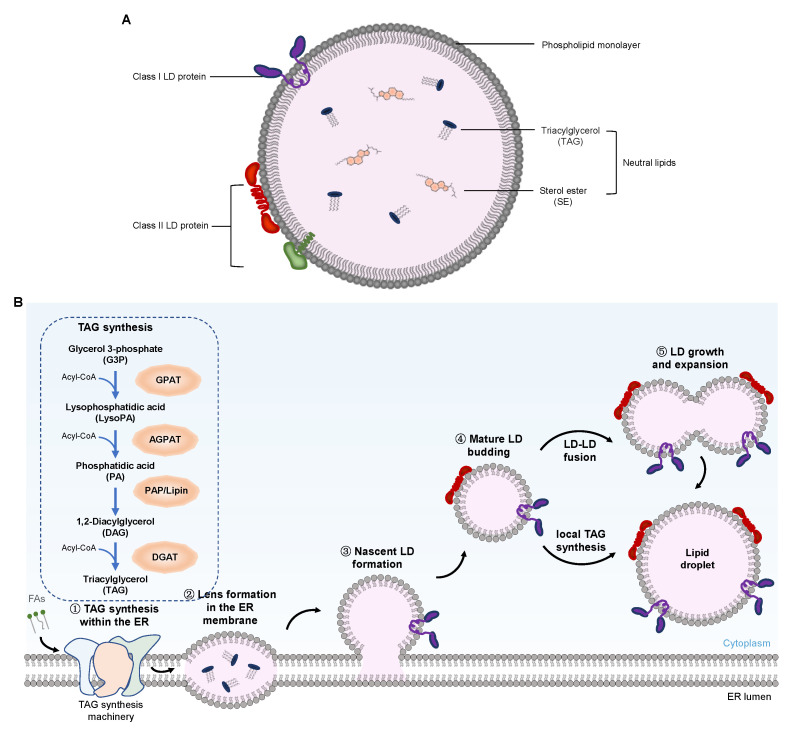
Schematic representation of the LD structure and a model of the key steps in LD biogenesis. (**A**) LD is composed of a core of neutral lipids, such as TAGs and SEs, surrounded by a phospholipid monolayer. LD surface-bound proteins are classified into two groups. Class I LD proteins, such as GPAT2, mostly contain a hydrophobic hairpin that is associated with the lipid monolayer; Class II LD proteins, such as perilipins, are inserted into the LD from the cytosol via amphipathic helices or other short hydrophobic domains. (**B**) Five essential steps are involved in LD formation, growth, and expansion. Abbreviations: LD, lipid droplet; TAG, triacylglycerol; SE, sterol esters; FAs, fatty acids; Acyl-CoA, acyl-coenzyme A; ER, endoplasmic reticulum; GPAT, glycerol-3-phosphate acyltransferase; AGPAT, acyl-glycerol-3-phosphate acyltransferase; PAP, phosphatidate phosphatase; DGAT, diacylglycerol acyltransferase.

The mature LDs mainly consist of a hydrophobic core composed of neutral lipids and a monolayer surface composed of phospholipids (Figure 2A). The hydrophobic core of LDs contains more than 100 different types of neutral lipids [27,28,29,30], which, in adipose and liver cells, are mainly composed of triacylglycerol (TAG) and cholesterol or sterol ester (SE). The phospholipid monolayer wrapping the hydrophobic core also has a unique composition of fatty acids (FAs) [31]. For LDs in mammalian cells, PC is the main component, accounting for approximately 60% of the phospholipid monolayer membrane. The changes in the ratios of phospholipids in the membrane affect the synthesis, maturity, and degradation of LDs [32,33].

The surface of LDs has several integration and peripheral proteins, which are divided into class I and class II proteins. Class II proteins are mainly from the PAT family, which is composed of five proteins in humans, namely, perilipin (also known as perilipin 1 or PLIN1), adipose-differentiation related protein (ADRP, also known as PLIN2), the 47 kDa tail interaction protein (TIP47, also named PLIN3), PLIN4. and PLIN5 [34,35,36,37]. These LD surface-bound proteins stabilize LDs and regulate the access of lipase and other enzymes to the neutral lipids in LDs. They also accumulate and control the size and interaction of LDs with other cellular organelles and the accessibility to lipolysis. In addition to PLINs, other LD proteins include enzymes participating in TAG and phospholipid synthesis or lipid transport.

LDs are mostly spherical and range from 50 to 200 µm in size. The specific LD size depends mainly on the state and type of the cells [36,38,39]. The underlining mechanism of LD regulation mainly involves cellular TAG contents or LDs fusion pathways. The latter determines the size of the LDs in mammary epithelial cells (MECs), as shown through live-cell imaging using a confocal fluorescence microscope. The degree of LDs fusion depends on the composition of phospholipids in the cell membrane and is enhanced by increased PE content, which also increases the size of LDs [40]. However, whether the mechanism of LDs, as regulated by the fusion pathway, has broad adaptability in different cell types requires further investigation on a more experimental basis.

### 2.2. Lipid Droplet Biogenesis

Several studies have been conducted in the past few decades to elucidate LD biogenesis (Figure 2B) [41,42,43,44]. Traditionally, LDs were only present in the cytoplasm of eukaryotic cells and were known as cytoplasm LDs (cLDs). It was believed that LDs originated from the ER, where the enzymes for the generation of neutral lipids are located [25,45]. The generation of these neutral lipids in the ER and their behavior in the membrane drive LD biogenesis. The LD formation begins with the accumulation of neutral lipids, such as TAG and SE molecules, which are synthesized by diacylglycerol acyltransferase 1 (DGAT1) in the ER between two leaflets of its membrane [32,46]. Because the two layers of ER phospholipids can only accommodate a small number of neutral lipids, once the concentration of neutral lipids in the double layers exceeds the critical point, the formation of LDs will be triggered. In fact, when the nascent LDs were formed in yeast, a lens of about 50 nm was observed in the ER [47]. As the nascent LDs grow, they will emerge (or bud) from the ER and separate [25], indicating that LDs seem to be formed spontaneously from the ER. This spontaneous bud mechanism does not require energy-consuming machinery, curvature induction reagents, or the intrinsic asymmetry of double layers [44]. Enzymes are necessary to synthesize neutral lipids in the process of LDs’ spontaneous budding, while proteins transform mature LDs, which process involves structural changes and the adjustment of LD biogenesis [25,48]. These proteins include PLIN3, glycerol-3-phosphate acyltransferase 4 (GPAT4), DGAT1, DGAT2, seipin, and fat storage-inducing transmembrane protein 2 (FIT2) [25,41,42]. Seipins are located at the ER–LD contact site, which directly affects the occurrence of LD biogenesis or is involved in regulating lipid metabolism [49,50,51,52,53]. The function of FIT is not completely clear, but the overexpression of FIT2 increases the size and number of LDs. The depletion of FIT2 causes the nascent LDs not to bud from ER but to still be embedded in the ER membrane, indicating that FIT regulates the budding of LDs from the ER. FITs may not directly mediate the budding but may affect the lipid homeostasis at the sites of LD biogenesis [54,55]. Molecular dynamic studies have shown that the protein recruited onto the ER membrane will be expelled precisely from the sites of LD formation, due to changes in the underlying membrane properties [48]. Lipids such as diacylglycerol (DAG) and phosphatidic acid also contribute to the formation of LDs by promoting shape changes in the same direction as the membrane curvature formation [25,56,57,58]. In the case of viral infection, the occurring process of LD biogenesis is observed and reflected by comparing their dynamics in virus-infected or uninfected cells. The data also demonstrate that LDs may be formed by peripheral TAG accumulation on the ER membrane and that HCV NS5A is responsible for interacting with those sites on the ER membrane that may form LDs [59].

Extensive research on LD biogenesis, carried out over several years, showed the important role ER plays in the growth of nascent and mature LDs. However, the hypothesis that LDs can spontaneously form from a symmetric elongated lens of ER membrane remains controversial [57], with some key questions, such as the location of the sites formed by LDs in ER, not yet having been answered. Other evidence suggests that LD formation in yeast and mammalian cells might occur at a unique ER site [60,61] with unknown characteristics, or it may be a process that occurs in random positions. Although the protein participating in TAG synthesis could be synthesized or enriched at the local discrete zones where LDs may be generated [62], most of the enzymes participating in neutral lipids synthesis are distributed throughout the ER, indicating that certain lipids may not only be generated at the site of LD assembly. Alternatively, neutral lipid synthase may be activated at the sites of LD biogenesis, while the lipin homolog, known as phosphatidic acid phosphohydrolase 1 (Pah1p) in yeast, is a regulator of lipid synthesis and may be positioned at the location of LD biogenesis in the ER. Though seipin and FIT2 may play a role in the sites of LD biogenesis [52,55,58], the way in which they regulate LD biogenesis in the ER remains to be clarified. When the nascent LDs transition to mature LDs, another question is whether they are separated from ER physically or are reconnected to the ER through the membrane bridge, whether they move from the cell periphery to the nucleus, and, if these bridges do exist, how can they form? In *Saccharomyces cerevisiae*, membrane bridge formation may not be required, given that the LD is still connected to the ER [63]. There also seems to be a unique mechanism in mammalian cells that uses the coat protein I (COPI) and adenosine diphosphate (ADP)–ribosylation factor 1 (ARF1) machinery to connect with the LDs separated from ER [64]. The answers to these questions will help us understand the machinery of LD biogenesis in the cell and elucidate the LDs’ interactions with other cellular organelles.

Recent evidence also shows the existence of nuclear LDs (nLDs), which originate from the inner nuclear membrane (INM) and are associated with INM via the predicted transmembrane protein, seipin [65,66]. Seipin forms a complex with LD assembly factor 1 (LDAF1) in the ER and plays a key role in cLD maturation [67]. The formation of nLDs is also related to promyelocytic leukemia (PML) nuclear bodies, which are dot structures in the nuclear matrix that regulates transcription and apoptosis in response to cell stress, including antiviral defense [68,69]. Since they were only found recently, their role has not yet been fully described, but, as with cLDs, nLDs may act as a lipid supplier for membrane expansion and as a bracket platform for proteins [70,71].

Besides nLDs, the secretory autophagosome released from DENV-infected cells also contains LDs. These extracellular LDs (eLDs) may involve the mechanism of cLD extracellular trafficking, which has not yet been thoroughly studied, but may be related to viral pathogenesis and could play an important role in the extracellular environment related to virus infection [72]. Whether the eLDs promote the early innate defense of DENV or other flaviviruses or are hijacked to spread the virus still needs further study.

Given the recent discovery of more types of LDs, their biogenesis should also depend on their positioning. In particular, the presence of nLDs indicates that LDs may also exist in the mitochondria derived from the endosymbionts. Therefore, it is necessary to determine whether they exist in other intracellular organelles or extracellular vesicles in eukaryotic cells. Theoretically, this speculation is reasonable because conservative LDs form a stable connection with ER and are also related to mitochondria, the inner nuclear envelope, lysosomes, vesicles, and endosomes [73,74].

### 2.3. Lipid Droplet Function and Its Regulation Mechanism

The LD is a highly complex organelle that stores energy and participates in the lipid metabolism of cells by acting as a hub for FA transport to the mitochondria. The LD also regulates nuclear functions through the availability of proteins and signaling lipids in the nucleus and protects cells from different forms of cellular stress, such as ER stress, and the lipotoxic effects of unesterified lipids [36]. In addition, LDs contribute to the maturity, degradation, storage, and turnover of many different proteins and maintain protein homeostasis [26,73,75]. Here, the main features of LD function are briefly described in the following sections.

#### 2.3.1. Participating in Cell Energy Storage and Lipid Metabolism

As an essential cellular organelle that regulates lipids and energy metabolism in cells, LDs are involved in many of the processes of cells, including lipid metabolism, membrane biogenesis, membrane transportation, and signal transduction. During nutritional stress, the lipids released from LDs are used either for hormone synthesis or as the primary repository of sterols, FAs, cholesterol, and the membrane phospholipid precursors used for cell membrane formation. They are used to maintain the homeostasis of ER and other membranes [47,76,77,78]. The lipids in the hydrophobic core of LDs are mostly TAG and SE, which play an important role in cell energy storage [36].

In cell lipid metabolism, a key metabolic pivot involves the regulation of phospholipid acid (PA) in the ER membrane, which is used to synthesize membrane phospholipids or LD TAGs. In yeast, PA is converted into DAG, a precursor of TAG, by PAH1. When nutrition is lacking, the PAH1 pathway is activated through the Nem1-Spo7 complex, which transfers the fatty acyl chain from PA to membrane biogenesis and keeps it stored in the LDs [58,79]. This may be an important part of cell survival reaction and the lipid flux-balancing mechanism that causes the LDs to allow cellular regulation into membranes for growth or storage, and for starvation/stress survival.

**Figure 3:**
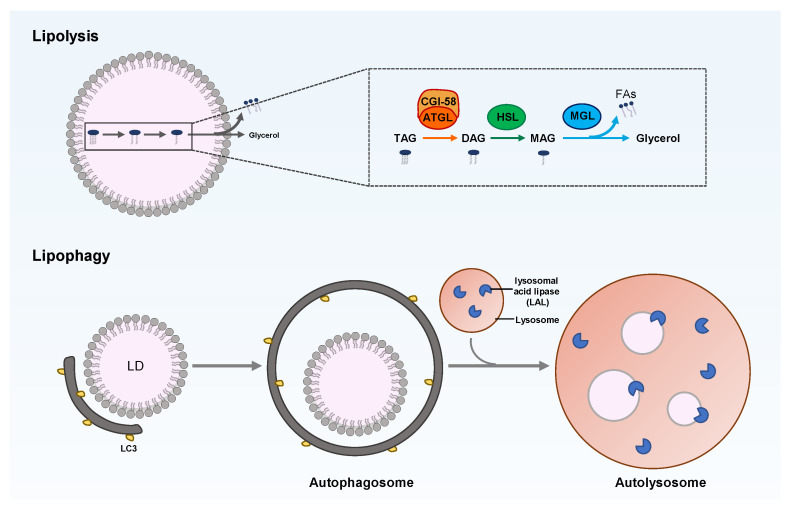
Overview of LD degradation through lipolysis or lipophagy. TAG breakdown in LD is catalyzed by cytoplasmic or lysosomal lipase. Lipolysis is composed of three sequential catalyzations of TAG, DAG, and MAG by adipose triglyceride lipase (ATGL), hormone-sensitive lipase (HSL), and monoacylglycerol lipase (MGL), respectively. Lipophagy belongs to selective autophagy; that is, the LDs are engulfed into the autophagosome and are then fused with lysosomes to form autolysosomes, wherein the neutral lipids in LDs are hydrolyzed by lysosomal acid lipase (LAL).

Cellular lipid metabolism is a complex and highly regulated process. The neutral lipids stored in LDs can be catabolized by cytoplasm lipolysis or lysosome-mediated lipophagy, which is the selective autophagy of LDs (Figure 3). Lipolysis is mediated by LD-related lipases, such as adipose triglyceride lipase (ATGL), while lipophagy involves the key enzymes that hydrolyze TAG and cholesterol in the lysosome, namely, lysosomal acid lipase (LAL), which acts on the LDs delivered to autolysosomes via autophagy.

Various proteins and lipases on the surfaces of LDs regulate LD catabolism. For example, perilipin is responsible for lipid homeostasis by controlling the availability of lipases to neutral lipids in LDs. At the basic level, perilipin combines with the comparative gene identification-58 (CGI-58) in an inactive state. Under starvation conditions, perilipin is phosphorylated by protein kinase A, releasing the combined CGI-58, which then combines with ATGL to drive the hydrolysis of TAG [80]. The phosphorylated perilipin can also add phosphate to the hormone-sensitive lipase (HSL) and transfer it to LDs. The HSL hydrolyzes DAG into monoacylglycerol (MAG) [81], which is hydrolyzed into free FAs and glycerol by monoacylglycerol lipase (MGL) [82]. The free FAs released by neutral lipid breakdown undergo β-oxidation to generate energy via mitochondria or the peroxisome [83]. Therefore, LDs play a key role in maintaining cellular homeostasis by balancing lipogenesis and lipolysis. This homeostatic effect is essential for normal cellular and organismal functions, which are closely related to human health. LD disorders can also cause many human diseases, such as fatty liver disease, obesity, diabetes, cardiovascular disease, and cancer [84,85].

#### 2.3.2. Preventing ER Stress

ER stress is caused by the activation of the unfolded protein response (UPR) by cells in response to conditions such as the aggregation of misfolded and unfolded proteins in the lumen of ER, the dysregulation of calcium homeostasis, and/or lipid composition imbalance, and the reaction process to re-establish ER homeostasis [86]. For example, three ER-integrated membrane proteins, also known as UPR sensors, are often activated in response to the accumulation of unfolded proteins. These proteins are the inositol-requiring protein 1 (IRE1), protein kinase RNA (PKR)-like ER kinase (PERK, also known as EIF2AK3), and the activated transcription factor 6 (ATF6). The activation of these three main pathways will trigger the cell signaling cascade, slowing the translation of related proteins and increasing the gene expression participating in ER protein-folding, degradation, and lipid biogenesis.

The disruption of LD biogenesis and TAGs synthesis often leads to UPR activation in yeast and mammalian cells. Studies on the mutant yeast strain (LDΔ) that is deficient in the enzymes required for TAG and SE biosynthesis with the loss of LDs showed an altered ER morphology. The alteration, however, could be reversed by inhibiting *de novo* FA synthesis or deleting the Opi1 inhibitor to induce ER phospholipid synthesis, suggesting that phospholipid synthesis could compensate for the loss of LD formation. Without LDs, the ER phospholipid composition changes, displaying an increased level of phosphatidylinositol, leading to impaired autophagosome biogenesis [78]. Although LDs function primarily by providing a lipid source to the autophagosome membrane [76,78,87], mutant yeast studies suggest that LDs are more likely to regulate autophagy through ER quality-control functions. Yeast cells with PC biosynthesis inhibition activate the UPR to form abnormally fragmented ER aggregates [88]. Excessive saturated FA triggers UPR more effectively than unsaturated FA [89,90]. However, UPR triggering does not require the unfolded protein-sensing domains of IRE1 and PERK and may involve sensing perturbed ER membrane composition [91]. In adipocytes, the lipolysis of stored lipids is stimulated by a noradrenergic signaling cascade, releasing large amounts of FAs, followed by the production of large numbers of small LDs, which are not fragmented or in fission with LDs, as initially thought [92,93]. Instead, these FAs are re-esterified and packaged into DGAT1-dependent LDs [94]. Under these lipolytic conditions, DGAT1-dependent fatty acid re-esterification and LD biogenesis are critical for protection against ER stress, lipotoxicity, and UPR activation. Furthermore, in 3T3-L1 preadipocytes, the lack of RAB18 resulted in a substantial reduction in the number of mature LDs and UPR activation, indicating increased ER stress after oleate treatment [95].

The molecular mechanism between impaired LD biogenesis and UPR activation is unclear. Because the abundance of LDs is usually found to increase when UPR is activated, an investigation needs to determine whether LDs improve or exacerbate this stress response. Studies also show that increased TAG storage in LDs mitigates ER stress in mammalian cells and tissues. The TAG storage of the liver LDs increases in ATGL-knockout mice, and the ER stress induced by the tunicamycin (TM) is prevented by reduced stress markers, such as the glucose-regulating protein 78 (GRP78) and the slicing X-box binding protein 1 (XBP1) [96]. Similarly, in human cardiomyocyte-derived cells, the overexpression of ATGL promotes the release of free FAs from the TAG in LDs, thereby increasing the ER stress markers. The ER stress induced by palmitate is also inhibited by increased TAG, through the overexpression of peroxisome proliferator-activated receptor γ (PPARγ) or acyl-CoA synthetase (ACSL1) [97]. The overexpression of autophagy-related 14 (ATG14) in Hela cells stimulates lipophagy, leading to increased free FAs and ER stress [98]. The constitutive hypoxia-inducible factor 2α (HIF-2α) upregulates the expression of the LD coat protein PLIN2 in clear-cell renal cell carcinoma (ccRCC). The knockdown of PLIN2, however, eliminates LD, expands the ER, and activates PERK, IRE-1α, and other UPR targets. Nevertheless, the supplies of the exogenous PLIN2 are sufficient to restore LDs and inhibit cell death [99].

The inhibition of PC biosynthesis in yeast cells activates UPR and forms LDs at the abnormal fragmented ER aggregates. The polyubiquitinated proteins and the ER chaperone heat-shock protein (Hsp104p) contained in LDs appear to be degraded via the endosomal sorting complex required for transport (ESCRT)-dependent microlipophagy in the yeast vacuole cells [88]. Data also indicate that LDs formed by an ER sequester unfolded, misfolded, or aggregated ER proteins and further promoted their removal through microlipophagy. However, whether these ubiquitinated proteins are located on the surface of LDs or on LD-related ER subdomains is unclear. The specific substrates involved in the assumed degradation pathways have not yet been determined. In mammalian cells, we observed a connection between LDs and selected ER-associated degradation (ERAD) substrates. Hydroxymethylglutaryl (HMG)-CoA reductase enzyme and apolipoprotein B (ApoB) undergo ERAD under specific metabolic conditions [100,101]. Interestingly, both proteins bind with LDs or are related to the LD-associated ER subdomains before degradation. In addition, changes in the lipid composition of the ER can also directly activate the UPR without requiring the luminal sensing domains of yeast and mammalian IRE1s and PERKs. Consistent with this finding, the activation of PERK and IRE1 by saturated FAs in recombinant liposomes requires that their transmembrane domains should be independent of the luminal misfolded protein-sensing domains [91,102]. These studies suggest that abnormal FAs storage in LDs can either activate UPR directly by changing the lipid composition of the ER membrane or indirectly, by changing ER homeostasis, leading to impaired ER protein-folding or calcium storage. The LDs provide a vehicle for rebalancing ER lipid homeostasis and removing misfolded ER proteins. This LDs-mediated function for ER quality control is likely to play a role, together with the ERAD pathway [103,104,105].

#### 2.3.3. Participating in Autophagy

Intracellular LDs are degraded into free FAs and glycerol in the form of autophagy, called lipophagy. It is manifested as the co-localization of autophagic LC3 and LD coat proteins, such as PLIN2. This kind of selective autophagy is another form of lipolysis and is necessary for the clearance of LDs and TAG in hepatocytes [106]. In addition to acting as an energy source, lipophagy controls the quality of LD proteins and lipid homeostasis in cells [107,108].

The occurrence of lipophagy mainly starts from the recognition of cargoes by the autophagosome membrane via interaction with the microtubule-associated protein 1 light chain 3 (MAP1LC3) [109]. This usually involves the assistance of one or more cargo adapters, such as P62, Optineurin, NBR1, and NDP52, which connect the organelle membrane to LC3. This has been confirmed by Tatsumi’s study [110] on the role of lipophagy in embryonic development. The study showed that the forced lipophagy system, through the fusion of the LD binding domain and p62, significantly reduced the number of LDs and TAG levels during mouse embryonic development, ultimately retarding the development. Meanwhile, lipophagy may also require the polyubiquitination of organelle surface proteins as a recruitment signal [111].

The LDs’ surface proteins involved in lipophagy and promoting LD recognition are not fully understood. Before autophagy or cytosolic lipase degrades LDs, the perilipin on the surface of LDs needs to be removed, which mainly occurs through chaperone-mediated autophagy, regulated by adenosine 5′-monophosphate (AMP)-activated protein kinase (AMPK) signaling [112,113]. Studies also show that huntingtin is necessary for lipophagy under stress conditions since it connects p62 with LC3-II, releasing Unc-51-like autophagy-activating kinase 1 (ULK1), which is a pro-autophagic kinase inhibited by mammalian target of rapamycin (mTOR) [114]. The ancient ubiquitous protein 1 (AUP1) is a type-III membrane protein and is expressed most commonly in LDs and the ER. It recruits the E2 ubiquitin-binding enzyme G2, accumulating LDs and controlling ER protein quality [115]. AUP1 is used by DENV and ZIKV to trigger lipophagy and may be a specific factor of lipophagy, but the mechanism of the initiation of lipophagy has not been fully elucidated. DENV deubiquitinates and transports AUP1 from LDs to autophagosomes by binding the viral NS4A and NS4B proteins in infected cells. DENV also stimulates the acyltransferase activity of AUP1 to upregulate lipophagy and promote the generation of progeny viruses. The knockdown of AUP1 using the clustered, regularly interspaced short palindromic repeats (CRISPR)-Cas9 genome editing technique eliminates the generation of the infectious DENV and ZIKV virion [116].

Proteins of the Rab molecular switch family may also be involved in the process of lipophagy. Many Rab molecules have been identified on LDs [117], some of which are related to autophagy regulation, especially Rab7, Rab10, and Rab25. They have been proven to be essential for lipophagy of hepatocytes under certain conditions. For example, Rab7 is activated in hepatocytes under nutrient deficiency; however, during lipophagy, it promotes the recruitment of multivesicular bodies and lysosomes to the LD surface. The deletion of Rab7 causes morphological changes in multivesicular bodies, lysosomes, and autophagosomes, and leads to reduced lipophagy [118]. In addition, chronic alcohol exposure inhibits the activation of Rab7, leading to the damage of lysosomes that degrade LDs and ultimately block the lipophagy of hepatocytes [119,120]. Rab10 forms a complex with the adaptor protein EH domain-binding protein 1 (EHBP1), while the membrane-modified adenosine triphosphatase EH-Domain Containing 2 (EHD2) to promote the migration of LC3-positive autophagic membrane to the LD surface. However, the loss of Rab10 function leads to LD accumulation [121]. The disappearance of LDs often accompanies the activation of hepatic stellate cells (HSCs). The activation of HSC stimulates the expression of Rab25 and promotes the formation of complexes with PI3KCIII, thereby guiding autophagy to recognize, encapsulate, and degrade LDs. The knockdown of Rab25 expression with specific siRNA, however, prevents lipophagy and inhibits the disappearance of LDs [122].

Lipase recognizes LDs and directly promotes the formation of autophagosomes by inducing the recruitment of TAG and SEs, which help to initiate lipophagy [76,123]. The cytoplasmic ATGL (also known as PNPLA2) regulates lipolysis and lipophagy, but the way in which it coordinates the regulation of both processes is unclear. ATGL is a necessary and sufficient positive regulator for inducing lipophagy in mouse liver. It interacts with LC3 via its LC3-interacting region (LIR) motif to promote its movement to LDs, upon which it causes lipophagy [124]. ATGL also promotes lipophagy by enhancing sirtuin 1 (SIRT1) activity, which regulates the catabolism of liver LDs [125]. The patatin-like phospholipase-domain-containing enzyme 5 (PNPLA5) that is present in LDs contributes to lipophagy and autophagic proteolysis [87]. PNPLA8 mediates the lipophagy driven by sterol-regulatory element binding protein 2 (SREBP-2) through its dynamic interaction with LC3 in the hepatocytes of high-fat diet (HFD)-fed mice and regulates lipid homeostasis in patients with nonalcoholic fatty liver disease (NAFLD) [126]. Through mutation studies, PNPLA3 was confirmed as a key player in the autophagosome formation stage of the lipophagy process in starved human hepatocyte HepG2 [127]. In lysosomes, LDs can also be degraded by acidic lipases. Under nutrient deficiency, the expression of lysosomal lipases in *Caenorhabditis elegans* and mouse hepatocytes is regulated by the lysosomal biogenesis transcription factor EB (TFEB) [128]. TFE3 regulates the steatosis of hepatocytes by inducing lipophagy [129]. Under fasting conditions, the forkhead homeobox transcription factor (FoxO1) triggers the lipophagy of adipocytes by inducing lysosomal acid lipase [130].

Similar to general autophagy, lipophagy is also regulated by cellular nutritional state-sensing signals and response systems, to ensure that the level of free FAs in cells matches their energy requirements. These sensory systems include AMPK, mTOR, the nuclear receptor farnesoid X receptor (FXR), PPARα, and transcription activator cAMP response element-binding protein (CREB) [120,131,132,133]. If the cells are in a nutrient-rich state and do not need free FAs as an energy source, lipophagy will be inhibited. However, in the case of insufficient nutrition, lipophagy is triggered, leading to the decomposition of TAG in LDs. The SIRT3 protein activates lipophagy by stimulating the AMPK-ULK1 pathway, inducing smaller-sized LDs, and reducing lipid accumulation in mature adipocytes [134]. For example, DENV induces AMPK kinase activity as well as AMPK-independent mTORC1 suppression to cause the lipophagy that promotes viral infection [135]. In addition, treatment with AMPK and an autophagy activator (AICAR and rapamycin) or mTOR inhibitor (Torin-1) significantly alleviates the symptoms in patients with NAFLD [136,137,138], indicating that restoring the function of lipophagy may be an important treatment for improving fatty liver disease.

## 3. Roles of LDs in the ZIKV Life Cycle

### 3.1. ZIKV Infection Causes Changes in LDs

To confirm that LDs participate in ZIKV infection, LDs are stained with specific dyes, such as BODIPY, Oil Red O, LipidTox Deep Red, and Nile Red, and then observed under a microscope to detect their intracellular changes after viral infection. To ensure that the effects of LD measurement occur in a single cycle of ZIKV replication, a low multiplicity of infection (MOI), i.e., infection with ZIKV at an MOI of 0.1 and a limited virus replication cycle (i.e., 24 h) are used for LDs analysis. Confocal microscopy showed that compared with uninfected cells, the number, content, and size of LDs in ZIKV-infected cells were significantly reduced [10]. This result is consistent with the previous findings that flavivirus infection induces pro-viral selective autophagy against LDs [139,140].

However, some studies have also reported increased LDs during the early stages of ZIKV infection. For example, two hours after ZIKV infection, both in vivo and in vitro, the induced LDs were transiently upregulated and controlled by the epidermal growth factor receptor (EGFR). The inhibition of EGFR suppresses the expression of LDs during infection, while the production of types I and III IFN, which reflects the ability to mount an effective immune response in infected cells, is significantly reduced. This ultimately leads to increased ZIKV replication, indicating that LDs can also serve as an important cellular organelle in the innate antiviral immune response [141,142]. In the model of infected astrocytes, cells were infected with ZIKV or stimulated with poly I:C, a dsRNA virus mimic. The LD displacement and average velocity in cells were significantly enhanced 2 h after infection or stimulation, indicating that the dynamics of LDs had changed in the early stage of pathogen infection, further supporting the emerging role of LDs in the innate host response [143]. Another study showed that hydroxysteroid (17β) dehydrogenase (HSD17B)12 promotes the replication of ZIKV and the production of infectious particles by increasing LD biosynthesis, which plays a key role in virus assembly. This study confirms the indispensability of LDs in ZIKV infection [144].

No explanation exists for the differences seen in LD changes following ZIKV infection. However, the differences may be due to the different cell systems or ratios of the virus to cells adapted to infect the monolayer cells [23,139]. Other reasons may be the research purposes used to determine the various analysis and measurement targets under corresponding experimental conditions. For example, some authors may pay more attention to the changes in a single LD or the number or total area of LDs, while others may only focus on the number of LDs. Given that the size of LDs may vary, the observed phenotype of LD changes may correspond to the overall activation of different cellular processes. The differences come from the various cell lines and the time of infection used. In various cell systems, viruses may adopt different LD hijacking paradigms. For example, ZIKV hijacks LDs by manipulating the SREBP pathway, which is the main regulator of LD biogenesis, or via secreted autophagosomes containing LDs [72,145]. There is also the possibility that the virus adopts different LD utilization modes at the various stages of infection. Therefore, in the early stages of viral infection, the virus may induce LD biogenesis to stimulate the initial replication and may then trigger lipophagy to reduce the number of LDs, to release free FAs from these lipid structures.

### 3.2. Interaction of LDs with ZIKV Proteins

Similar to other flaviviruses, the ZIKV-C protein is also one of the viral structural proteins assembled with the viral genome to form the nucleocapsid. In addition to the classical structural function that accommodates and protects the virus genome, the C-protein also has multi-functional characteristics. It plays an important role in viral replication and infection by interacting with cellular proteins and regulating cell metabolism, apoptosis, and immune response.

**Figure 4:**
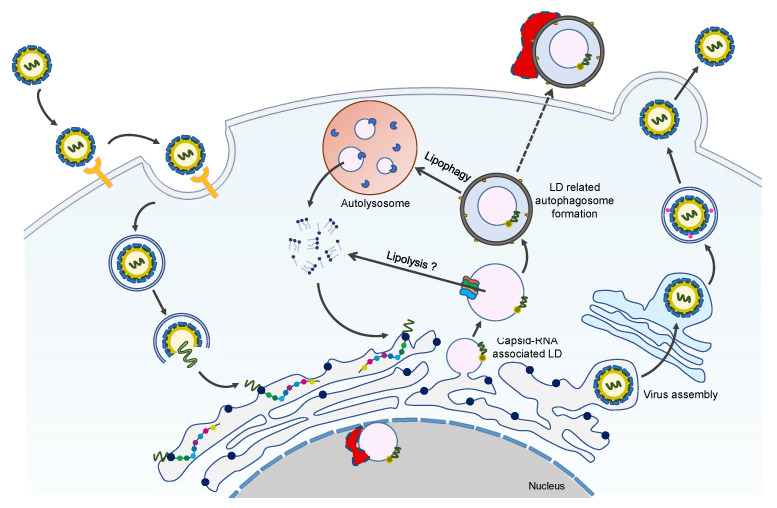
Overview of LDs-C protein interaction during the ZIKV life cycle. ZIKV infection promotes LDs formation at the early stages of infection. With the increasing interaction of LDs with ZIKV-C protein, lipophagy is induced to generate sufficient free fatty acids for robust viral replication and/or assembly. During this process, ZIKV NS4A/4B proteins may also contribute to the occurrence of lipophagy. We hypothesize that the eLDs and nLDs are involved in the ZIKV life cycle, and their formation and function possibly need the help of some unknown host factors, which are shown as red irregular polygons with a border of dashed lines.

Previous studies have shown that the ZIKV-C protein is localized in the LDs of the cells in infected HEK293, BHK21, and Vero [15,16,22], representing the primary location of the C-protein in host cells. This is also consistent with DENV and HCV-C protein localization in the LDs [23,146]. The interaction between the C-protein and host LDs is important for viral genome encapsulation, replication, and assembly (Figure 4). In addition, the accumulation of C-protein around the LD structure is the key prerequisite for the efficient production of viral particles [147,148].

Shang et al. [22] resolved the crystal structure of the ZIKV C protein with a resolution of 1.9 Å. The structure contains four α helices with a unique long pre-α1 loop, contributing to C-C dimer formation. Compared with the known forms of WNV and DENV-C proteins, the ZIKV-C protein has different hydrophobic characteristics at the lipid bilayer interface. The interaction between the ZIKV-C protein and LDs was confirmed by confocal microscopy analysis. The substitution mutation of key amino acids (F27S/K31S/R32S) in the pre-α1 loop of ZIKV-C protein disrupted the interaction with LDs, indicating that this loop is critical for membrane association [22].

The dynamic N-terminal domain of the C-protein is related to its functional diversity. A specific peptide (pep14–23) in the DENV-C protein mediates the binding of the C-protein to LDs through conformational transition [149]. The characteristics of the structural dynamics of the N-terminal domain of the ZIKV-C protein were analyzed by dividing the domain into three truncated fragments. The circular dichroism, dynamic light scattering, Zeta potential, and molecular dynamic simulation confirmed that the truncated fragments, 5–26 and 1–30, are prone to adopt an α-helical conformation, but the conserved fragment, 14–23, is unstructured. This fragment does not undergo conformational conversion, which is different from the DENV-C protein, suggesting that this conserved region in the ZIKV-C protein may not be involved in the association with LDs [150]. The molecular mechanism of the interaction between ZIKV-C protein and LDs needs further study.

In addition to the interaction of ZIKV-C protein with LDs, ZIKV NS4A and NS4B proteins are also associated with the selective autophagy of LDs. DENV NS4A and NS4B proteins induce lipophagy to improve the production of infectious particles by interacting with AUP1 to hijack its acyltransferase function. This mechanism seems also to play a role in ZIKV infection and is likely to be a universal mechanism adopted by flavivirus infection [116].

## 4. Conclusions

Lipid droplets serve as a platform for ZIKV replication and a lipid reservoir for cells and provide energy and lipid sources for virus replication. By comparing the size, location, accumulation, and dynamic changes of LDs through real-time visualization techniques in uninfected and infected cells, we reveal the important details of how ZIKV hijacks cellular LDs for its successful replication and understand in what stages of the virus life cycle the LDs may play a role. However, the molecular mechanism of LD involvement in ZIKV infection still needs further elucidation. How ZIKV establishes contact, interacts, and communicates with cellular LDs needs to be answered. Identifying and characterizing viral factors (such as structural and NS proteins) and host factors, especially the specific LD-related factors involved in the interaction between ZIKV and LDs, is also important. Identifying and characterizing these interaction factors can provide basic information for developing effective therapeutic drugs that inhibit ZIKV replication. In recent years, with the improvement of the isolation and purification of LDs from cultured cells, the morphology, lipidomics, proteomics, and even “ZIKV-LDs interaction omics” after viral infection will be characterized in depth. The data will provide important information for further understanding the role of LDs in the pathogenesis of flaviviruses.

Since there are no effective antiviral drugs for ZIKV, developing antiviral drugs is still challenging. In this sense, positioning lipid metabolism regulators as antiviral drugs is a promising strategy for the future. Existing studies also support the possibility of developing antiviral treatment strategies based on LDs, a basic and conserved cellular organelle. For example, the use of SREBP pathway inhibitors (such as PF-429242) to control LD abundance and change LD size and proteome has been proven to be an attractive option for controlling flavivirus infection [145,151,152]. However, this lipid metabolism antiviral drug has a long way to go before it can be applied in clinical practice.

Therefore, a comprehensive understanding of the important role of LDs in the life cycle of ZIKV helps to understand the pathogenic mechanism of viruses and may make them possible targets for developing new antiviral therapies.

## Data Availability

Not applicable.
